# Supramolecular polymerization of a prebiotic nucleoside provides insights into the creation of sequence-controlled polymers

**DOI:** 10.1038/srep18891

**Published:** 2016-01-04

**Authors:** Jun Wang, Peter V. Bonnesen, E. Rangel, E. Vallejo, Ariadna Sanchez-Castillo, H. James Cleaves II, Arthur P. Baddorf, Bobby G. Sumpter, Minghu Pan, Petro Maksymovych, Miguel Fuentes-Cabrera

**Affiliations:** 1 Center for Nanophase Materials Sciences, Oak Ridge National Laboratory, TN 37831, USA; 2 Computer Science and Mathematics Division, Oak Ridge National Laboratory, TN 37831, USA; 3 Earth-Life Science Institute (ELSI), Tokyo Institute of Technology, 2-12-1-IE-1, Ookayama, Meguro-ku, Tokyo, 152-8550, Japan; 4 Institute for Advanced Study, 1 Einstein Drive, Princeton, NJ 08540; 5 Blue Marble Space Institute of Science, 1515 Gallatin St. NW, Washington, DC 20011; 6 Center for Chemical Evolution, Georgia Institute of Technology, Atlanta, GA 30332, USA; 7 Escuela Superior de Apan, Universidad Autónoma del Estado de Hidalgo, Carretera Apan-Calpulalpan km. 8, Apan Hidalgo, C.P. 43900, México; 8 MOE Key Laboratory of Fundamental Physical Quantities Measurements, School of Physics, Huazhong University of Science and Technology, Wuhan, 430074, China

## Abstract

Self-assembly of a nucleoside on Au(111) was studied to ascertain whether polymerization on well-defined substrates constitutes a promising approach for making sequence-controlled polymers. Scanning tunneling microscopy and density functional theory were used to investigate the self-assembly on Au(111) of (*RS*)-N^9^-(2,3-dihydroxypropyl)adenine (DHPA), a plausibly prebiotic nucleoside analog of adenosine. It is found that DHPA molecules self-assemble into a hydrogen-bonded polymer that grows almost exclusively along the herringbone reconstruction pattern, has a two component sequence that is repeated over hundreds of nanometers, and is erasable with electron-induced excitation. Although the sequence is simple, more complicated ones are envisioned if two or more nucleoside types are combined. Because polymerization occurs on a substrate in a dry environment, the success of each combination can be gauged with high-resolution imaging and accurate modeling techniques. These characteristics make nucleoside self-assembly on a substrate an attractive approach for designing sequence-controlled polymers. Further, by choosing plausibly prebiotic nucleosides, insights may be provided into how nature created the first sequence-controlled polymers capable of storing information. Such insights, in turn, can inspire new ways of synthesizing sequence-controlled polymers.

Interest in sequence-controlled polymers, *i.e.* polymers composed of two or more monomers that are arranged in a specific sequence, is growing steadily, fueled in part by their potential applications in information storage and catalysis, and generally by the fact that controlling the sequence could lead to enhanced control of polymer structure and function^1^. Synthesizing sequence-controlled polymers is however quite challenging. Two approaches are typically used to control the monomer sequence in a polymer^2^. One employs biological concepts; the other uses chain-, step- and multistep-growth polymerization. The latter approach was recently shown to enable the incorporation of monomers into a sequence with a very high precision^3^, the encoding of sequences which can be read and erased^4^, and the creation of synthetic sequences containing more than a hundred monomers^5^. Polymerization on well-defined surfaces in dry environments, constitutes a viable third approach that could mitigate some of the limitations of wet-lab protocols, namely low yield, lack of control, and their time-consuming nature^6^.

Polymerization on a substrate has several interesting features that are attractive for synthesizing sequence-controlled polymers. On a substrate, polymers can be studied in detail using high precision imaging techniques, such as scanning tunneling microscopy (STM), which in turn can be used to read a sequence. Because self-assembly on a substrate is the result of a subtle interplay between substrate and molecule interactions, changing the substrate is often used to control how the molecules self-assemble, a property that can lead to polymerization in chosen locations. Polymers supported on substrates can also undergo chemical and structural transformations when subjected to external stimuli^7^,^8^, which might allow error correction or even encoding of new sequences (*e.g.* erasable and re-writable memory storage). Finally, on a substrate, the polymer is usually more amenable to modeling with accurate techniques, such as density functional theory (DFT), which permits fine details on the electronic, vibrational and structural properties of the self-assembled polymer to be obtained.

In a series of very interesting works, Hecht and Grill and co-workers have shown how self-assembly on a substrate can be used to create covalently-bonded one- and two-dimensional molecular structures in a precise manner. In their approach, molecules with groups that are reactive are deposited on a substrate^9^. When the temperature is raised, reaction occurs and leads to the formation of polymers and membrane-like structures. The polymers can be a 100 nm long, can be pulled off the substrate with a STM tip, can be made to grow at precise locations on the substrate, and can be composed of a backbone with side-chains that come out at regular intervals^10^,^11^. Here, however, we focus on molecules that can self-assemble into hydrogen-bonded polymers instead of covalently-bonded ones. The reason being that it is convenient to create sequence-controlled polymers that can be formed in a reversible manner, so that a sequence can be both written and erased in a precise location and time via the application of an external stimulus, i.e. voltage across the STM tip. Nucleosides molecules fulfill these requirements.

Nucleosides are composed of two linked moieties, a nucleobase and a sugar. When a nucleoside is deposited on a substrate, the nucleobase and the sugar interact with each other via hydrogen bonds and with the substrate via van der Waals interactions. This leads to supramolecular self-assembly. Yang *et al.*^12^ recently showed that thymidine, a nucleoside composed of thymine (T) and 2-deoxyribose (D), self-assembles on Au(111) into chains and islands that, when subjected to consecutive scans of the STM tip, transform into exquisitely organized 4-nm long parallel-lines. Each line is comprised of a hydrogen-bonded polymer where T only binds to T and D to D. This polymer has a sequence, albeit a very simple one, *i.e.* T-D-D-T, etc. More complicated sequences can be envisioned by designing nucleosides with moieties that bind to others in a preferential manner.

Research on the substrate-supported self-assembly of nucleosides can also provide valuable insights into how nature created the first sequence-controlled polymers. In fact, the notion that nucleobases can self-assemble in a variety of ways when deposited on substrates has often been raised in self-assembly scenarios that might have contributed to the formation of the first informational oligomer in pre-biotic Earth. However, in this context, research on substrate-supported self-assembly of nucleosides remains, surprisingly, largely unexplored.

In this work we use STM and DFT to investigate the self-assembly of (*RS*)-N^9^-(2,3-dihydroxypropyl)adenine (DHPA) on Au(111). It is shown that DHPA self-assembles into a hydrogen-bonded polymer that grows almost exclusively along the herringbone reconstruction pattern of Au(111), encodes a sequence that is maintained intact for hundreds of nanometers, and is erasable by applying voltage across the STM tip. DHPA is one of the nucleosides in Glycol Nucleic Acid^13^, an oligomer whose structural simplicity and stability makes it a plausible candidate for a pre-RNA informational oligomer. The observation that DHPA forms an extended polymer raises the possibility that, on weakly interacting substrates, a plausible prebiotic nucleoside might self-assemble more efficiently than the natural ones.

## Results

### STM characterization of the supramolecular polymer

**Figure 1:**
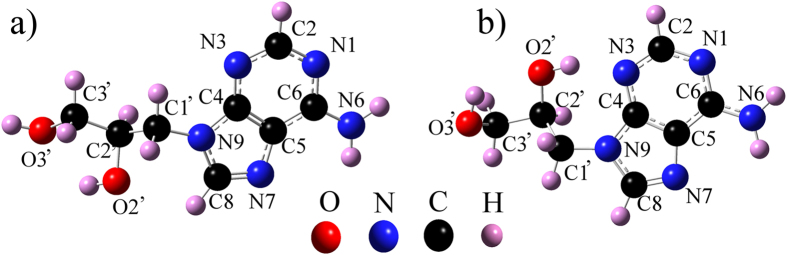
Two different conformers of the *S*-enantiomer of DHPA. (**a**) Conf1. (**b**) Conf2. Conf2, which DFT found to be 217 meV more stable than Conf1, has an internal hydrogen bond which connects the OH group of C2’ to the N3 atom of the adenine moiety.

DHPA, shown in Fig. 1, is a nucleoside analog of adenosine and is composed of a 2,3-dihydroxypropyl moiety (hereafter referred to as the “glycol” group) connected to adenine at its *N*9 position. The sample used in this study was purchased from Sigma-Aldrich and, as it was part of Aldrich’s Rare Chemical Library, no analytical data was available. NMR and circular dichroism were used to verify its identity and determine its level of purity. Comparison of the ^1^H NMR spectrum in DMSO-*d*_6_ with literature data^14^ revealed the compound to be (*RS*)-N^9^-(2,3-dihydroxypropyl)adenine in ≥98% purity. Analysis of a 0.1 mg/mL solution in distilled deionized (Milli-Q) water by circular dichroism (Jasco J-810, 0.1 mm path length cell, 190–310 nm scan) revealed the compound to be a racemic mixture^15^ of the *R*- and *S*-enantiomers (DHPA has a chiral center at the C2’ position, see Fig. 1).

**Figure 2:**
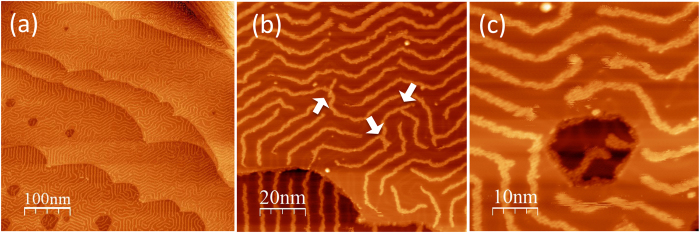
STM images of self-assembled structures made of DHPA on Au(111). (**a**) Chains meander on Au(111), growing mainly on the herringbone reconstruction pattern and extending over terraces; (**b**) the chains occasionally branch out and produce Y-junctions, indicated by the white arrows; (**c**) the chains growing inside the nano pit in areas where the herringbone reconstruction is still present. Scanning conditions: (**a**) Sample bias U = 1 V, tunneling current I = 50 pA, temperature T = 104 K, (**b,c**) U = 0.8 V, I = 50 pA, T = 104 K.

*In situ* vacuum thermal evaporation of DHPA on Au(111) led to the self-assembly of polymer chains. Figure 2 shows constant current STM images captured at liquid nitrogen temperature: the chains are at least 100 nm long, grow mainly along the herringbone reconstruction pattern of Au(111), and branch out occasionally in Y-shaped junctions (indicated by arrows in Fig. 2b). The average spacing between the chains is ~ 6 nm, which is the spacing between the two neighboring *hcp* reconstructed regions on Au(111). The herringbone reconstruction thus guides DHPA self-assembly. This is supported by the observation that the chains grow inside the dark-hexagonal pits on the Au(111) terraces (Fig. 2c). These pits, made by sputtering a clean Au(111) surface, were created to investigate whether step edges facilitate self-assembly. Although chains are found at the step edges of the pits, they grow predominantly in areas where the herringbone pattern is present.

**Figure 3:**
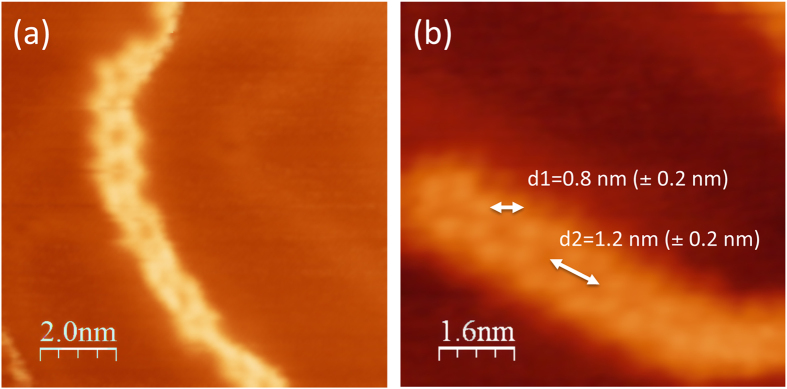
STM images show DHPA double-chains on Au(111) with molecular resolution, revealing these chains consist of hexagonal-like structural motifs. Scanning conditions: (**a**) U = 0.8 V, I = 50 pA, T = 141 K; (**b**) U = −0.1 V, I = 100 pA, T = 103 K.

The STM image in Fig. 3a shows that each chain consists of two interconnected polymeric chains forming a repeating hexagonal-like motif. The high-resolution STM images in Fig. 3b show that the hexagonal motif consists of six individual protrusions, each the size of a single DHPA molecule. The nearest distance between two molecules, referred to as d1, is ~ 0.8 (±0.2) nm, while the distance between the ring center of two neighboring hexagons, d2, is ~ 1.2 (±0.2) nm.

### DFT development of a model for the polymer

DFT calculations were used to develop a model of the supramolecular DHPA polymer. The relative stability of the conformational isomers of DHPA, *i.e.* Conf1 and Conf2 of Fig. 1, was first investigated in gas-phase. Conf2 was found to be 217 meV more stable than Conf1. As a consequence, from now on we focus on Conf2 only. The binding strength of Conf2 to the Au(111) surface was investigated by considering three different configurations: top, bridge and fcc, characterized by having O3’ located on top of a Au atom of the first layer, midway between two Au atoms of the first layer, and on top of a Au atom of the third layer, respectively; in all three configurations, O3’ is pointing towards the surface. Binding energies determined from DFT are given in Table 1. Without the van der Waals (vdW) interactions, the top configuration is the most stable one, followed by bridge and fcc. In the top configuration, DHPA is bound to Au(111) with an energy of 0.127 eV and with O3’ at 3.3 Å vertically away from the Au atom below. If vdW interactions are included, the binding energy increases to 1.048 eV and the most stable structure is fcc, with O3’ at 3.1 Å from Au. Adenine binds to Au(111) with an energy of 0.1 eV (0.9 eV if vdW corrections are included) and has its ring center at 3.5 Å (3.3 Å with vdW)^16^ vertically away from Au. The energetics and relative distance of DHPA and adenine on Au(111) are thus very similar. From now on it will be assumed (as it was also done for adenine monolayers on Au(111)^17^) that the hydrogen bonding interactions, and not the interactions with the substrate, dominate the polymeric self-assembly of DHPA on Au(111). Further, as for each configuration of DHPA on Au(111) both enantiomers bind with similar strength, it can be concluded that the polymer contains both enantiomers. The question now is, what is the structure of this polymer and has it been observed before in the context of nucleobase self-assembly?

A very similar structure has been observed for adenine before, albeit on Cu(111). Furukawa *et al.*^18^ and Shinoda *et al.*^19^ found that adenine molecules deposited on Cu(111) self-assemble into a polymeric structure that they called a double-chain. The double-chain resembles the polymer observed here: both are composed of two chains that are interconnected and form a hexagonal-like motif, however the adenine double-chain is at most 10 nm long. We created a model of the DHPA polymer following the same prescription Shinoda *et al.*^14^ used to develop a model for the adenine double-chain^13^. This prescription consists of investigating the stability of dimers in gas-phase and using the dimers, like pieces of a puzzle, to build a hydrogen-bonded polymer.

**Figure 4:**
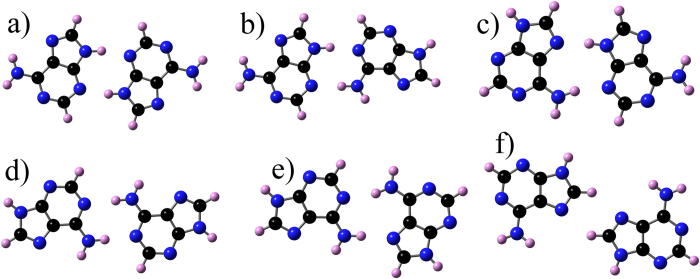
Dimers of the adenine molecule. (**a–e**): dimers I to V; Furukawa *et al.*^18^ and Shinoda *et al.*^19^ used these dimers to develop a model for single- and double-chains made of adenine on Cu(111); (**f**) A6A6: a dimer that could potentially be a link between the two single-chains in the adenine double-chain structures termed as DCH2 and DCH3 by Shinoda *et al.*^19^.

**Figure 5:**
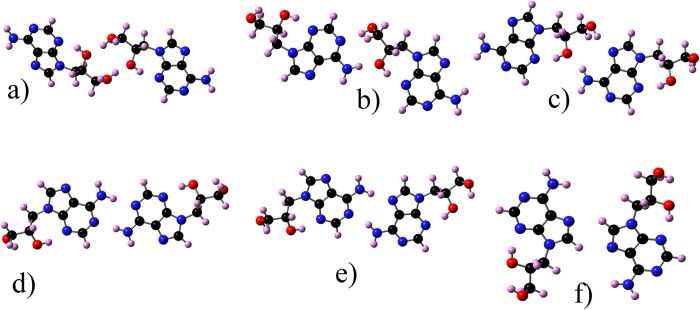
Dimers of the DHPA molecule. (**a–e**): dimers I to V used to develop a model for single- and double-chains made of DHPA molecules; (**f**) A6A6: a dimer that links the two single-chains in the DHPA double-chain model.

**Figure 6:**
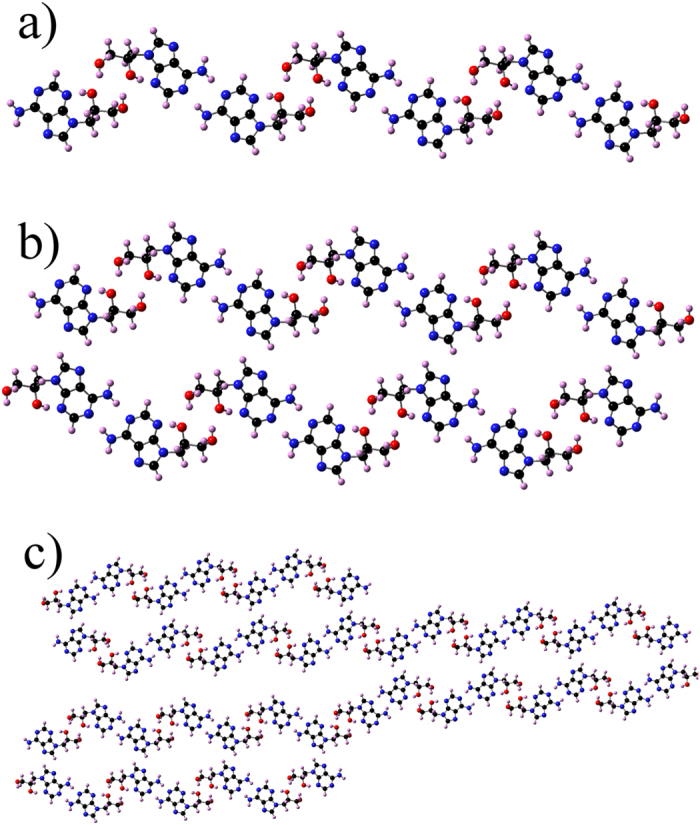
DFT models developed in this work to explain the self-assembled DHPA molecular chains. (**a**) Single-chain; (**b**) double-chain; (**c**) Y-junction. All three models are made of Conf2 *R*-enantiomers only. All the models are held by hydrogen-bonds that connect the adenine and glycol moieties among themselves.

Adenine and corresponding DHPA dimers are shown in Fig. 4 and Fig. 5, respectively, while their corresponding stabilities are shown in Table 2. The DHPA dimers IV and V are as stable as their adenine counterparts, which is due to the fact that in these dimers binding is mediated by the adenine bases only. When bonds that involve the glycol group hold dimers, as in the case of DHPA dimers I, II and III, the stability is significantly reduced. (This is due to the large value of the deformation energies, see Supplementary Table S1.) Adenine double-chain models were developed imposing three conditions^14^. First, the most stable dimer, in this case dimer I, is the basic unit of each single-chain. Second, consecutive I’s are connected by the same two hydrogen bonds along a single-chain. Third, a double-chain is made by two parallel single-chains. This led to a single-chain where I is flanked on both sides by either IV or V, which are the less stable adenine dimers. The double-chain was made by connecting two parallel single-chains by the bond A6A6 (for this bond we use the same nomenclature used by Kelly *et al.*^20^); the structure and relative stability of A6A6 are shown in Fig. 4f and Table 2, respectively. In the case of DHPA, dimer A6A6, see Fig. 5f, has a similar stability to its adenine counterpart (not surprising given the fact that this dimer is also held by the interaction between the adenine moieties), dimers IV and V are the most stable ones, and dimer I is the least stable. Despite these differences, the same three conditions can still be applied for building a polymeric model for a single-chain by flanking a DHPA dimer IV with dimer I, Fig. 6a, and a DHPA double-chain by connecting two single-chains via the A6A6 bond, Fig. 6b. The resultant hydrogen-bonded polymer contains the same sequence in each of its chains, namely glycol-glycol-adenine-adenine-glycol-glycol, etc.

This DHPA polymeric model agrees well with the experimental observations: it accommodates different enantiomers (see Supplementary structural parameters for the unit cells of DHPA double-chains containing different amounts of *S*- and *R*-enantiomers), in agreement with the racemic nature of the sample; its d1 and d2 are 1.0 and 1.5 nm, reasonably close to the measured 0.8 (±0.2) nm and 1.2 (±0.2) nm; and, as illustrated in Fig. 6c, explains Y-junctions as the confluence of three DHPA double-chains.

### STM controlled nanoscale manipulation of the supramolecular polymer

**Figure 7:**
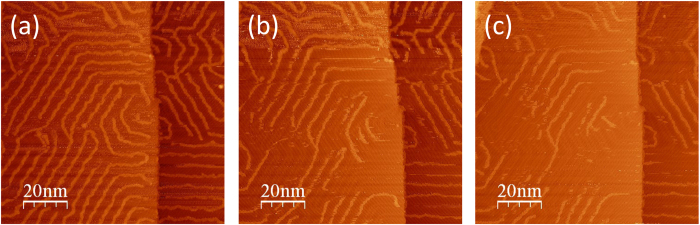
Three STM images obtained consecutively at the same location, which show that a change of tunneling bias or scan direction can erase the DHPA double-chain structure. The meandering DHPA chains in (**a**) are partially erased in (**b**) and about halfway through erased in (**c**), revealing some intact gold herringbone structures originally under the DHPA chains. Scanning conditions: (**a**) U = 0.9 V, I = 100 pA, T = 141 K; (**b**) U = −0.8 V, I = 500 pA, T = 141 K; (**c**) U = −0.8 V, I = 500 pA, T = 141 K.

To realize the idea of manipulating these sequence-controlled polymers to enable “erasing and re-writing”, we demonstrate that sections of the polymer can be erased in a controlled fashion by applying an external stimulus. The sample was subjected to a bias perturbation through the STM tip to investigate possible chemical and structural transformations. Figure 7 shows three images that were taken consecutively as the bias changed. Figure 7a shows the initial structure taken at a sample bias of 0.9 V; the polymers are shown spread evenly throughout two Au terraces and covering the entire herringbone patterns. Figure 7b was taken subsequently and at a sample bias of −0.8 V. The polymers have partially disappeared revealing the Au herringbone pattern intact underneath. This is even more obvious in Fig. 7c, which was obtained subsequently using a different time frame and scan direction. A perturbation of bias is therefore able to “erase” the polymer.

## Discussion

Self-assembly of DHPA on Au(111) was studied to explore if supramolecular polymerization in well-defined substrates is a promising approach for designing sequence-controlled polymers. STM images revealed that DHPA self-assembles into a polymer made of two interconnected chains, producing a structure that is similar to that made by adenine molecules on Cu(111). Density functional theory was used to develop a model of the DHPA polymer, and in the process it was found that DHPA dimers where adenine moieties bind to each other are as stable as counterpart adenine dimers; by contrast, DHPA dimers where a glycol binds to glycol or to adenine are significantly less stable. Combining the most and least stable DHPA dimers, a method that has been used before for explaining self-assembly patterns of adenine on Cu(111)^14^ and Au(111)^12^, led to a model for a DHPA double-chain hydrogen-bonded polymer with characteristics that agree well with the experimental observations. This polymer differs from that made by thymidine on Au(111)^7^ in three significant aspects: the DHPA polymer appears spontaneously and not as a consequence of an external stimulus, it is at least 2 orders of magnitude longer (the actual length surpasses the range of the STM apparatus), and it grows almost exclusively along the herringbone reconstruction pattern.

The reason for the increased length of the supramolecular polymer is likely associated with the strength of the interactions that hold it together. The glycol-glycol interaction is 0.19 eV smaller than the adenine-adenine interaction, while in the thymidine polymer D-D is 0.46 eV less stable than T-T^7^. This suggests that nucleoside- or nucleoside analogs-based hydrogen-bonded polymers could be made longer if the interactions that hold them together have a similar strength. It is also noteworthy that the extra propensity for longer chains comes from the templating effect of the herringbone reconstruction. The latter can be due to non-uniform strain or electrostatics of the surface^21^. Whatever the case, additional confinement of the longer chains to the quasi-1D directions along the hcp-stacked regions may help reduce the three-fold degeneracy dictated by the symmetry of the pristine (unreconstructed) surface lattice and skew the growth toward longer oriented chains in favor of shorter chains with more equivalent orientations. This alone points to an interesting pathway for sequence-controlled polymerization on nanoscale template substrates.

Although the polymer found here has a very simple sequence, *i.e.* glycol-glycol-adenine-adenine-glycol-glycol that is repeated over long distances, more complicated sequences can be envisioned by simply combining DHPA with any of the other four nucleosides that comprise GNA, or other besides. The challenge here resides in ensuring that the chosen nucleosides pair-up in a way that produces the desired sequence. For example, if one chooses DHPA and (*RS*)-N^9^-(2,3-dihydroxypropyl)thymine (DHPT), the challenge is to polymerize them in the sequence glycol-adenine-thymine-glycol-etc. For this purpose, DFT studies become invaluable. As shown above, DHPA dimers that pair up via the adenine moieties have the same stability as the corresponding adenine dimers. There is no reason to believe that this would not be true for dimers made of other GNA nucleosides. This means that to determining whether a particular pair of GNA nucleosides pairs up in the desired manner, one can look up the stability of the corresponding nucleobase dimers in the existing DFT databases^22^. Alternatively, one could use glycol-based Janus-nucleosides to produce such sequences. Recently, He and collaborators synthesized Janus-nucleosides composed of AT, and these were found to form supramolecular structures in an aqueous environment^23^. Conceivably Janus-nucleosides with glycol groups could also be synthesized. Clearly, the range of possibilities is staggering, and the fact that the polymers are grown on a substrate permits combining high-throughput DFT-based techniques (as those developed by Lukas *et al.*^12^ to predict all the possible monolayer structures that adenine makes on Au(111)), synthesis and STM imaging to gauge and optimize selected nucleosides. That DHPA self-assembly only occurs along the herringbone reconstruction pattern of Au(111) is also very exciting in the context of information technology. For example, if one assumes that a sequence encodes a sentence, different sentences could be combined into a paragraph. Enforcing polymerization on parallel patterned-lines on a substrate could create each of these sentences. Finally, if self-assembly were to produce undesired structures, such as the Y-junctions observed here, these can be erased with a STM tip.

All these characteristics make substrate-supported polymerization of nucleosides a very promising approach for creating sequence-controlled polymers. Interestingly, this approach also provides insights into how nature might have created the first sequence-controlled polymers capable of storing information. In particular, the findings here indicate that on substrates that existed on early Earth and interacted weakly with supported molecules, GNA nucleosides could have self-assembled better than current DNA/RNA nucleosides. This might not be a unique characteristic of GNA nucleosides, and thus investigating the substrate self-assembly of other plausible prebiotic nucleosides could very well inspire new ways of creating sequence-controlled polymers.

## Methods

### Experimental methods

STM experiments were conducted using a locally designed variable temperature (25 to 300 K) STM in an ultrahigh vacuum (UHV) chamber with a base pressure of 1 × 10^−10^ Torr. The Au(111) sample was cleaned via a standard sputtering and annealing process until scans showed large clean terraces. Hexagonal nano pits on Au(111) were created after a short gentle sputtering (1 kV, 1.0 × 10^−7^ Torr, for 1 minute) on a heated (~150 °C) sample. A commercial Pt-Ir tip (Agilent Technologies) was prepared by field emission treatments on a clean gold surface. DHPA powder was sublimed onto the substrate *in situ* via a physical vapor deposition process from a laboratory-built Knudsen cell. This *in situ* vacuum deposition technique proved to be suitable for large-scale molecular self-assembly in a controlled environment for STM characterization^24^. The vapor pressure stayed ~9 × 10^−9^ Torr during the deposition. A total deposition time of 15 minutes gave the coverage seen in the STM images. The sample was annealed at room temperature for about an hour before inserting it into the liquid nitrogen cooled STM. The bias voltage was applied on the sample and STM imaging was performed primarily at liquid nitrogen temperatures, ranging from 80–145 K.

### Theoretical methods

The NWChem^25^ suite of programs was used to perform DFT calculations in the gas-phase to investigate the stability of DHPA’s isomers and adenine and DHPA dimers. These calculations employed the B3LYP^26^,^27^ hybrid functional to approximate the exchange-correlation potential and the 6-31G** basis-set. The software VASP^28^ was used to relax with DFT the single- and double-chains structure in gas-phase and to compute the binding energy of Conf2 on the Au(111) surface. The calculations employed the Perdew, Burke and Ernzerhof approximation^29^ for the exchange correlation potential, the projector-augmented wave pseudopotentials^30^, and the vdW-DF2 correction to account for dispersion interactions^31^,^32^; depending on the size of the system 6 or 1 (Γ) k-points were used. A single DHPA molecule was placed on an Au(111) hexagonal substrate made of 144 atoms distributed in four layers. The substrate had x, y and z dimensions of 17.73, 17.73 and 31.23 Å, respectively. Each configuration was relaxed by allowing all the atoms to move, except for those of the fourth layer of Au. The relaxation was stopped when the forces on each atom were equal or smaller than 0.02 eV/Å. The double-chain structures were simulated with a monoclinic unit cell that contains two DHPA dimers. Several double-chain models were created, each containing a different amount of *R*- and *S*-enantiomers (see Supplementary). In particular double-chains with 100% *R*-enantiomers, 100% *S*-, 50% *R*- and 50% *S*-, were created and relaxed until the forces were equal or smaller than 0.02 eV/Å. The calculations were all carried out employing only the Γ-point and did not include the vdW interaction. The single-chain structure of Fig. 7a was created by deleting one of the chains in the double-chain of Fig. 7b, which was made of 100% *R*-enantiomers.

An initial geometry for a Y-shape structure with a length of 118.25 Å was first optimized with the semiempirical method PM3^33^,^34^. The resultant structure was then relaxed again with DFT as implemented in Gaussian 09 and employing the hyper-GGA B3LYP correlation-exchange functional with the triple zeta 6-311G(d,p) basis-set^26^,^27^,^35^,^36^.

## Additional Information

**How to cite this article**: Wang, J. *et al.* Supramolecular polymerization of a prebiotic nucleoside provides insights into the creation of sequence-controlled polymers. *Sci. Rep.* **6**, 18891; doi: 10.1038/srep18891 (2016).

## Supplementary Material

Supplementary Information

## Figures and Tables

**Table 1:** Binding energies (in eV) for Conf2 on Au(111).

Configuration	*R*-enantiomer	*S*-enantiomer
Top	0.127 (1.040)	0.128 (1.042)
Bridge	0.117 (1.039)	0.116 (1.039)
Fcc	0.112 (1.048)	0.107 (1.046)

The values in brackets were obtained with the vdW correction.

**Table 2:** Binding energies (in eV) for dimers of adenine and DHPA (see Fig. 4 and Fig. 5, respectively) corrected for the basis-set superposition error and deformation energies.

Type of dimer	ΔE^a^	ΔE^b^
I	−0.74 (−0.21^c^/−0.79^d^)	−0.29
II	−0.61 (−0.17^c^/−0.72^d^)	−0.41
III	−0.56 (−0.17^c^/−0.69^d^)	−0.38
IV	−0.50 (−0.14^c^/−0.60^d^)	−0.48
V	−0.45 (−0.13^c^/−0.57^d^)	−0.45
A6A6	−0.14 (−0.18^d^)	−0.13

^a^Binding energies for dimers of adenine.

^b^Binding energies for dimers of DHPA.

^c^Shinoda *et al.*^19^;

^d^Kelly *et al.*^20^.
