# Supplementary material for: Supramolecular polymerization of a prebiotic nucleoside provides insights into the creation of sequence-controlled polymers
Source: Sci Rep. 2016 Jan 4;6:18891. doi: 10.1038/srep18891 (PMC4698751; doi:10.1038/srep18891)
Supplement: Supplementary Information [file srep18891-s1.pdf]

Supplementary Information

Supramolecular polymerization of a prebiotic nucleoside provides insights into the creation of sequence-controlled polymers

Jun Wang,[†] Peter V. Bonnesen,[†] E. Rangel,[††] E. Vallejo,[††] Ariadna Sanchez-Castillo,[††] H. James Cleaves II,[‡,¶,#,||] Arthur P. Baddorf,[†] Bobby G. Sumpter,[†,§] Minghu Pan,[†] Petro Maksymovych,[†,*] and Miguel Fuentes-Cabrera[†,§,*]

[†]Center for Nanophase Materials Sciences, Oak Ridge National Laboratory, TN 37831, USA

[§]Computer Science and Mathematics Division, Oak Ridge National Laboratory, TN 37831, USA

[‡]Earth-Life Science Institute (ELSI), Tokyo Institute of Technology, 2-12-1-IE-1, Ookayama, Meguro-ku, Tokyo, 152-8550, Japan

[¶]Institute for Advanced Study, 1 Einstein Drive, Princeton, NJ 08540

[#]Blue Marble Space Institute of Science, 1515 Gallatin St. NW, Washington, DC 20011

[||]Center for Chemical Evolution, Georgia Institute of Technology, Atlanta, GA 30332, USA

[††]Escuela Superior de Apan, Universidad Autónoma del Estado de Hidalgo, Carretera Apan-Calpulalpan km. 8, Apan Hidalgo, C.P. 43900, México

*Corresponding author: maksymovychp@ornl.gov; fuentescabma@ornl.gov

Table S1 Adenine (DHPA) base pair energies (in eV). The DHPA dimers are made of the Con2 isomer only.

Type of dimer	E_{BSSE}	E_{def}	ΔE
I	-0.81 (-0.59)	0.068 (0.31)	-0.74 (-0.29)
II	-0.68 (-0.57)	0.069 (0.16)	-0.61 (-0.41)
III	-0.64 (-0.52)	0.077 (0.14)	-0.56 (-0.38)
IV	-0.55 (-0.53)	0.052 (0.049)	-0.50 (-0.48)
V	-0.51 (-0.51)	0.056 (0.055)	-0.45 (-0.45)
A6A6	-0.15 (-0.14)	0.004 (0.005)	-0.14 (0.13)

Table S2 Length and angle (in Å and deg) of the hydrogen bond in Adenine (DHPA) dimers. For the dimers I-III, the bonds in Adenine and DHPA dimers are not the same. The later are indicated in brackets.

Type of dimer	Hydrogen bond	Length	Angle
I	N9-H...N3 (O3'-H...O2')<br>N9-H...N3 (O3'-H...O2')	2.90 (2.76)<br>2.90 (2.75)	168.2 (165.3)<br>168.2 (164.5)
II	N6-H...N3 (N6-H...O2')<br>N9-H...N1 (O3'-H...N1)	3.00 (2.88)<br>2.90 (2.81)	177.9 (170.8)<br>169.7 (169.8)
III	N6-H...N3 (N6-H...O2')<br>N9-H...N7 (O3'-H...N7)	3.00 (2.93)<br>2.90 (2.79)	175.2 (161.1)<br>167.0 (162.5)
IV	N6-H...N1<br>N6-H...N1	3.00 (3.01)<br>3.00 (2.97)	178.8 (177.7)<br>178.8 (178.5)
V	N6-H...N1<br>N6-H...N7	3.00 (2.98)<br>3.00 (3.02)	175.9 (165.6)<br>166.2 (176.5)
A6A6	C8-H...N7<br>C8-H...N7	3.32 (3.33)<br>3.32 (3.33)	140.2 (140.2)<br>140.2 (140.2)

Lattice constant parameters of the monoclinic unit cells that were used to simulate DHPA double-chains in gas-phase. All the double-chains are made of Conf2.

Double-chain with 100% R-enantiomers:

A = 43.4694546000 Å

B/A = 0.4509372981 Å

C/A-ratio = 1.1809051738 Å

COS(β) = -0.9599083184

V = 12261.08 $Å^3$

Double-chain with 100% S-enantiomers:

A = 42.5130568179 Å

B/A = 0.4704437059

C/A = 1.1789379343

COS(β) = -0.9549996114

Volume = 12640.00 $Å^3$

Double-chain with 50% R- 50% S-enantiomers for each chain

A = 44.3520642135 Å

B/A = 0.4509372981

C/A = 1.1809051738

COS(β) = -0.9599083184

Volume = 13023.20 $Å^3$

Two single-chains made by 100% R- and 100% S-enantiomers

A = 44.3520642135 Å

B/A = 0.4509372981

C/A = 1.1809051738

COS(β) = -0.9599083184

Volume = 13023.20 $Å^3$
